# Speckle-Tracking Echocardiography in Dogs: Evaluating Imaging Parameters and Methodological Variability in Global Longitudinal Strain Assessment

**DOI:** 10.3390/ani15111523

**Published:** 2025-05-23

**Authors:** Jonas E. Mogensen, Maiken B. T. Bach, Pernille G. Bay, Tuğba Varlik, Jakob L. Willesen, Caroline H. Gleerup, Jørgen Koch

**Affiliations:** 1 Department of Veterinary Clinical Sciences, Faculty of Health and Medical Sciences, University of Copenhagen, Dyrlægevej 16, 1870 Frederiksberg, Denmarkmaiken.thode@sund.ku.dk (M.B.T.B.); jw@sund.ku.dk (J.L.W.); caroline.gleerup@sund.ku.dk (C.H.G.); 2 Department of Veterinary Medicine, Internal Medicine, Bursa Uludağ University, Görükle Kampüsü, Görükle, 16059 Nilüfer, Bursa, Türkiye; tugbavarlik777@gmail.com

**Keywords:** canine, speckle-tracking echocardiography, myocardial function, 2D-STE, global longitudinal strain, dog, heart

## Abstract

Two-dimensional speckle-tracking echocardiography (2D-STE) is an advanced imaging technique that offers quantitative insights into myocardial function by analyzing the motion of speckles created during ultrasound–tissue interactions. This study aims to evaluate the reliability of 2D-STE by examining the impact of key technical parameters on global longitudinal strain (GLS) measurement accuracy and comparing two speckle-tracking analysis methods provided by GE Healthcare: quantitative analysis of the 2D strain (2D strain) and automated function imaging (AFI). The prospective study consisted of two cohorts. In the first cohort, including 16 healthy dogs, the influence of frame rate, heart rate variation, zoom, transducer frequency, and image foreshortening on speckle-tracking values was assessed. In the second cohort, which included 10 healthy dogs, 2D-STE parameters were obtained with the 2D strain and AFI to assess agreement between the methods and observer variability. Our findings indicate that foreshortening (*p* < 0.01, Cohen’s d: 0.52, CI: −17.81 to −24.83) and heart rate variability (*p* = 0.02, Cohen’s d: 0.72, CI: −18.07 to −26.23) significantly affect speckle-tracking measurements. While zoom, frame rate, and frequency did not show a significant impact. Additionally, while the 2D strain and AFI exhibited a strong correlation, a significant systematic bias was identified, with AFI underestimating strain values compared to the 2D strain. Intra- and inter-observer coefficients of variation (CV) were below 9% for both methods, supporting their reliability. These findings emphasize the need to optimize image acquisition and selection criteria, which enhances the accuracy and reliability of the speckle-tracking analysis.

## Simple Summary

Two-dimensional speckle-tracking echocardiography (2D-STE) is a valuable tool for assessing myocardial function by measuring myocardial deformation. This prospective cohort study investigates 2D-STE. The first part of this study evaluated the influence of heart rate, frame rate, foreshortening, zoom, and transducer frequency upon global longitudinal strain (GLS) in 16 healthy dogs. The second part of the study compared GLS values obtained with GE Healthcare’s two 2D-STE methods: quantitative analysis of the 2D strain (2D strain) and automated function imaging (AFI) in 10 dogs. Our findings demonstrate that foreshortening of the left ventricle (*p* < 0.01, Cohen’s d: 0.52, CI: −17.81 to −24.83) and heart rate variation (*p* = 0.02, Cohen’s d: 0.72, CI: −18.07 to −26.23) significantly affect the GLS_A4C_ in dogs, which should be considered in clinical and research applications. There was a good correlation between the 2D strain and AFI-obtained 2D-STE parameters, as well as low interobserver variability (between an experienced and a novice observer). However, due to systemic bias, the 2D strain and AFI should not be used interchangeably.

## 1. Introduction

Two-dimensional speckle-tracking echocardiography (2D-STE) is an advanced imaging technique that quantitatively assesses myocardial function by analyzing the movement of naturally occurring acoustic speckles within ultrasound images. In human cardiology, 2D-STE has transitioned from an emerging non-invasive technique into an essential clinical tool for evaluating myocardial deformation and systolic function [1,2]. The development of standardized guidelines by the EACVI/ASE/Industry taskforce [3] has played a crucial role in promoting its widespread clinical adoption, improving both reproducibility and clinical utility. Among the strain parameters derived from 2D-STE, global longitudinal strain (GLS) has shown proven value due to its reproducibility and strong correlation with other methods for myocardial deformation [4,5]. The GLS measures myocardial shortening along the longitudinal axis and is typically obtained from three standardized apical views: apical four-chamber (A4C), apical two-chamber (A2C), and apical long-axis view (APLAX) [2,4]. To enhance consistency in interpretations, guidelines recommend using the absolute GLS to assess systolic function [3].

In veterinary medicine, 2D-STE has emerged as a promising modality for myocardial function assessment in both healthy and diseased dogs [6,7]. Several studies have aimed to establish reference values for GLS across different canine breeds [6,8,9,10] and investigate physiological conditions [9,11,12]. However, despite its potential, 2D-STE has not yet been widely integrated into routine veterinary clinical practice. This is mainly due to technical limitations, such as suboptimal image quality, variability in acquisition parameters, and differences in vendor-specific software implementations [3,13,14]. Among these challenges, four key imaging parameters have been identified as critical determinants of GLS accuracy: frame rate (FR), heart rate (HR), transducer frequency, and foreshortening of the apical views [3,4,15].

FR optimization is essential, as the inadequate temporal resolution can compromise speckle-tracking accuracy, leading to tracking artifacts and unreliable strain measurements [2,3]. In human echocardiography, an FR range between 40 and 100 frames per second (FPS) is recommended to ensure an optimal balance between temporal resolution and tracking fidelity [2,3,4]. Similarly, fluctuations in HR can affect GLS estimation, with higher HRs potentially leading to undersampling and underestimation of strain [16]. The transducer frequency also plays a significant role as it affects image resolution and tissue penetration depth. Higher transducer frequencies improve spatial resolution but may limit tissue penetration, whereas lower frequencies enhance penetration at the expense of speckle visibility [17].

Additionally, foreshortening of apical views remains a notable challenge in veterinary and human echocardiography, as improper image acquisition can artificially shorten the left ventricular long axis, leading to GLS overestimation [15,18]. Left ventricular ejection fraction by Simpson’s method is another systolic function technique affected by foreshortening [17,19]. As both ejection fraction and STE depend on left ventricle geometry, this emphasizes the need to investigate the effect of foreshortening on STE [5,19].

Beyond imaging parameters, vendor-specific differences in 2D-STE software contribute to variability in GLS measurements [3,20]. Notably, GE Healthcare offers two distinct 2D-STE methodologies: quantitative analysis for the 2D strain (2D strain) and automated function imaging (AFI). While both methods use speckle tracking, AFI is primarily designed for clinical applications, whereas the 2D strain is more commonly used in research settings. Comparative studies evaluating these methodologies in human cardiology are limited, and discrepancies in measurement algorithms may further impact the interpretation of strain value and clinical decision-making.

To address the knowledge gaps and improve the clinical reliability of 2D-STE in veterinary cardiology, this study aims to:

1. Evaluate how key imaging parameters (frame rate, heart rate, transducer frequency, and foreshortening) affect GLS measurements in clinically healthy dogs.
2. Compare the performance of GE Healthcare’s 2D strain and AFI in speckle-tracking echocardiography.

By systematically assessing these factors, we aim to enhance the clinical application of 2D-STE in veterinary cardiology, ensuring a more standardized and reproducible GLS assessment before broader clinical implementation in veterinary practice.

## 2. Materials and Methods

This study was conducted at the University Hospital for Companion Animals, University of Copenhagen, Denmark. The Ethical and Administrative Committee approved the study (EAU 2023-30). Data were prospectively collected from two cohorts designed to evaluate key aspects of 2D STE in dogs. The first cohort focused on imaging parameters, including FR, HR, transducer frequency, image zooming, and foreshortening on strain measurements. The second cohort aimed to compare the performance of the 2D strain and AFI methodologies, assessing intra- and inter-observer variabilities between an experienced veterinary cardiologist (JK) and a final-year veterinary student (JEM). Together, these two datasets contribute to a unified investigation into the technical factors influencing GLS measurement in veterinary cardiology.

### 2.1. Animals

**Figure 1:**
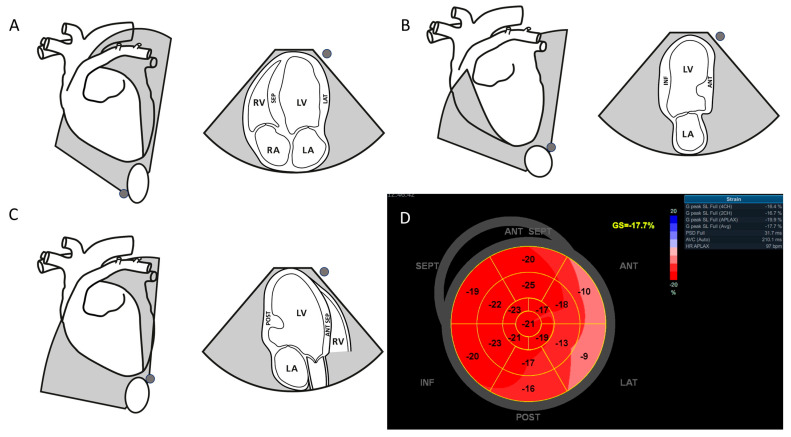
Schematic illustration of three left apical views and probe placement used to measure automated function imaging and quantitative analysis for 2D strain. (**A**) Illustrates an apical four-chamber view (septal and lateral wall); (**B**) Illustrates an apical two-chamber view (anterior and inferior wall); (**C**) Illustrates an apical long-axis view (posterior and anterior septal wall); (**D**) Global longitudinal strain (GLS) by speckle-tracking echocardiography. A Bullseye from a healthy dog with a GLS of −17.7%. The Bullseye shows regional longitudinal strain for each segment of a 17-segment left ventricle model. Bright red shows the most negative normal values of GLS. The grey circle represents the indicator placement of the ultrasound probe (**A**–**C**).

A total of 26 clinically healthy dogs were enrolled, with 16 dogs included in the first cohort between September 2023 and January 2024 and 10 dogs included in the second cohort between August and December 2024. All dogs were presented for cardiac screening at the University Hospital for Companion Animals. The dogs underwent a standardized cardiac evaluation, which included a clinical examination, electrocardiography (ECG), and transthoracic echocardiography, followed by apical acquisitions from the apical four-chamber (A4C), two-chamber (A2C), and long-axis (APLAX) views for speckle-tracking analysis (Figure 1). Echocardiographic examinations were performed by a single investigator (JK) on non-sedated animals in right and left lateral recumbency. Imaging was conducted using a GE Healthcare Vivid E95 Vet system (Brondby, Denmark) with M5SC-D, 6S, and 4Vc megahertz (MHz) multi-frequency phased-array transducers. All cine loops were digitally stored for subsequent offline analysis.

### 2.2. Speckle-Tracking Echocardiography

Speckle-tracking echocardiography was conducted by optimizing grayscale settings to enhance visualization of the endocardial borders, ensuring optimal tracking quality. For each dog, three-to-five consecutive cardiac cycles were recorded with simultaneous one-channel ECG to facilitate accurate timing of cardiac events. In the first cohort, echocardiographic acquisitions were systematically modified to evaluate the influence of various imaging parameters on global strain derived from A4C view (GLS_A4C_). Each dog underwent imaging under multiple conditions, beginning with an optimized A4C view (GLS_A4C-Zoom_) as the baseline reference. Additional acquisitions included a zoomed A4C view and an A4C view captured at FR, ranging from 53 to 112 frames per second (FPS) using a 6S MHz transducer. To assess the effects of higher temporal resolution, separate images were obtained at elevated FR, ranging from 128 to 193 FPS (GLS_A4C↑Frame rate_).

**Figure 2:**
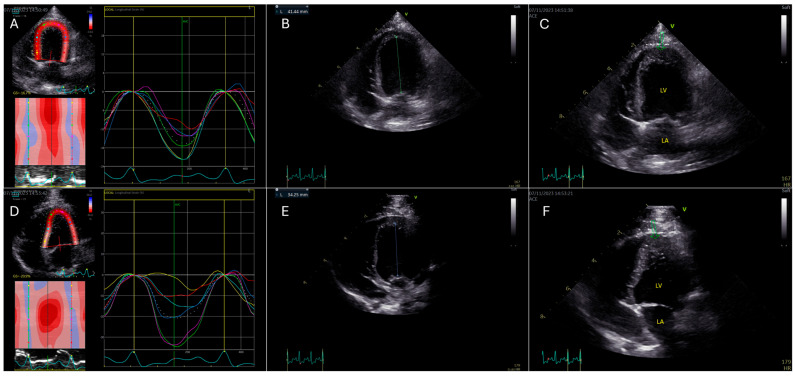
Assessment of global longitudinal strain (GLS) in an optimized versus a foreshortened apical four-chamber (A4C) view in a healthy miniature Bull Terrier using 2D speckle-tracking echocardiography (2D STE). (**A**): 2D STE trace from an optimized A4C view with appropriate apical imaging. The segmental longitudinal strain curves (colored lines) correspond to the six myocardial segments of the left ventricle in this view, illustrating synchronized myocardial shortening with a calculated GLS of −16.7%. (**B**): The optimized A4C view in end-diastole demonstrates a long-axis measurement from the mitral annulus to the apex of 41.44 mm. (**C**): Optimized A4C view in systole. The green arrow indicates the true apex, which appears smooth, pointed, and normally contracting, confirming the absence of foreshortening. (**D**): Foreshortened A4C view with altered image geometry. The 2D STE-derived strain curves (colored lines) show increased segmental strain values and a falsely elevated GLS of −20.9%, despite identical patient and imaging conditions. (**E**): Foreshortened A4C view in end-diastole, showing a reduced LV long-axis length of 34.25 mm, consistent with incomplete apical visualization. (**F**): Foreshortened A4C view in systole, where the apex (green arrow) appears thickened, blunted, and irregular, further confirming suboptimal acquisition.

Furthermore, to evaluate the impact of altered imaging planes, foreshortened A4C views were acquired by positioning the transducer one-to-two intercostal spaces cranial to the standard A4C imaging window (GLS_A4C-Foreshortening_) (Figure 2). HR manipulation was performed to assess its effect on GLS measurements; this was achieved by inducing mild excitement through auditory cues or treats, resulting in increased HR ranging from 167 to 222 beats per minute (GLS_A4C↑HR_). To examine the influence of transducer frequency, additional A4C views were obtained using lower frequency transducers, including the M5SC-D and 4Vc transducers (GE Medical, Brondby, Denmark), with frequencies ranging from 1.7/3.3 MHz to 2.3/4.6 MHz (GLS_A4C-Frequency_).

Acquisitions performed at elevated HR were consistently conducted last to prevent residual excitement or physiological recovery from interfering with measurements taken under standard conditions. This systematic approach allowed for a comprehensive evaluation of the impact of varying imaging parameters on GLS measurements, ensuring consistency and reliability in the data-collection process.

### 2.3. Offline Analysis, Brondby, Denmark

Offline analysis was conducted using EchoPac v. 204 (GE Healthcare). In the first cohort, analysis was performed exclusively using the 2D strain methodology, comparing strain values across different imaging conditions in the A4C view. In the second cohort, GLS obtained from A4C, A2C, and APLAX were analyzed using the 2D strain and AFI methodologies without mirror imaging, following the same standardized protocol across all dogs. The APLAX view was analyzed first for event timing of end-systole, followed by sequential analysis of the A4C and A2C views. End-systole was determined using pulsed-wave Doppler of the aortic flow obtained in the apical long-axis (APLAX) view, as described in the EACVI/ASE/industry task force consensus statement. End-diastole was set at the peak of the R-wave on the ECG, as used by the GE EchoPac software (version 204) and in accordance with the recommendations from the EACVI/ASE/industry task force. The region of interest (ROI) was manually adjusted to ensure complete myocardial tracking from the endocardium to the epicardium, excluding the pericardium and papillary muscles. Tracking quality was assessed visually, and adjustments to the ROI were made until optimal border delineation was achieved. Measurements with poor or insufficient image quality were excluded from the final analysis.

For intra- and inter-observer variability assessment, GLS was independently measured by a highly trained veterinary cardiologist and a final-year veterinary student. Repeated measurements were performed 1 week later using the same cine loops.

### 2.4. Statistical Analysis

Data analysis was performed using GraphPad Prism (ver. 10.1.1) and RStudio (ver. 2024.09.1 build 394) with statistical significance set at a *p*-value of <0.05. The normality of echocardiographic and demographic variables, including age and weight, was assessed using the Shapiro–Wilk test.

To evaluate the difference between the technical parameters and the two 2D-STE methods, GLS values were analyzed using a paired *t*-test for normally distributed data and a paired Mann–Whitney U test for non-normally distributed data. In the first cohort, the GLS_A4C_ values obtained from the optimized A4C view were set as the reference, against which longitudinal strain values from adjusted acquisition parameters were compared.

The correlation between GLS obtained by the 2D strain and AFI was assessed using the Pearson correlation coefficient for normally distributed data and the Spearman Rank correlation coefficient for non-normally distributed data. Correlation strength was categorized based on the classification by Schober et al. [21]: negligible (r = 0.00–0.10), weak (r = 0.10–0.39), moderate (r = 0.40–0.69), strong (r = 0.70–0.89), and very strong (r = 0.90–1.00). The limits of agreement between the two methods were visually examined using Bland–Altman plots. Agreement limits were defined as ±1.96 SD of the mean difference and were represented as distinct lines on the plots, together with the confidence interval for the mean difference. The intra- and inter-observer variability was quantified using the coefficients of variation (CV), calculated via the root-mean-square approach.

## 3. Results

A total of 26 clinically healthy dogs were included in the study, divided into two cohorts (Cohort 1: n = 16; Cohort 2: n = 10). Although minor incidental findings, such as physiological murmurs and intermittent second-degree atrioventricular block, were noted in some dogs, none were considered to impact strain analysis, and all patients were retained in the study. Descriptive statistics can be found in Table 1. All parameters followed a normal distribution, except for GLS_APLAX_ measured with AFI by the veterinary student in the first analysis and GLS_A4C_ measured with either AFI or the 2D strain in the A4C view by the trained cardiologist in the first analysis in cohort 2.

In cohort 1, the recorded images in nine dogs were inadequate for analysis in specific parameters due to poor image quality. Specifically, foreshortened images (GLS_Foreshortening_) could not be analyzed in three dogs, images with increased heart rate (GLS_A4C↑HR_) were inadequate in five dogs, and one dog had inadequate low-frequency images (GLS_A4C-Frequency_) (2).

GLS values from the A4C view were analyzed under different imaging conditions in cohort 1. Significant deviations from the baseline were observed for GLS_A4C-Foreshortening_ and GLS_A4C↑HR_, with increases in absolute GLS values of 11% and 15.4%, respectively (*p* < 0.01, Cohen’s d = 0.52, CI: −17.81 to −24.83), and *p* = 0.02, Cohen’s d = 0.72, CI: −18.07 to −26.23). No significant changes were noted for GLS_A4C-Zoom_, GLS _A4C↑Frame rate_, or GLS _A4C-Frequency_ (Table 2).

Cohort 2 found a strong correlation between the 2D strain and AFI in most left apical views. AFI consistently underestimated GLS values compared to the 2D strain, indicating a systematic bias (Table 3).

**Figure 3:**
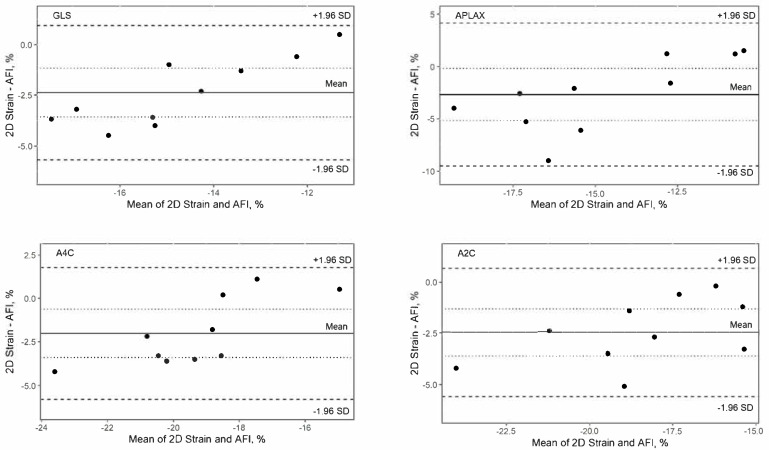
Bland–Altman plots comparing the 2D strain and AFI measurements. Solid line—mean difference between 2D strain and AFI, dashed lines—the limits of agreement, dotted lines—95% confidence intervals for the mean difference. The plots shown are GLS (**top left**), GLS_APLAX_ (**top right**), GLS_A4C_ (**bottom left**), and GLS_A2C_ (**bottom right**).

Furthermore, Bland–Altman plots found that the values mostly lie between the limits of agreement, but the mean difference lies below 0, supporting the finding of a systemic bias. Bland–Altman also revealed that larger strain values were associated with greater discrepancies in the cardiologist’s analyses, suggesting a proportional bias (Figure 3).

The intra- and inter-observer CV remained below 9% for all measurements, indicating good reproducibility [22,23] (Table 4). Notably, AFI demonstrated lower intra-observer variability in the cardiologist assessments, suggesting greater consistency could be achieved with AFI.

## 4. Discussion

This study investigated the impact of technical parameters on GLS measurements using 2D-STE in healthy dogs and examined the interchangeability of GLS values derived from the 2D strain and AFI. The findings provide new insights into how image-acquisition settings, using advanced echocardiography, influence myocardial deformation assessments and highlight the systemic biases that prevent direct comparability between different strain-analysis methods.

In the first cohort, GLS_A4C_ measurements were significantly affected by image foreshortening and increased HR, while changes in FR, image zoom, and transducer frequency did not produce statistically significant changes. The increase in absolute GLS_A4C_ values by 11% (*p* < 0.01) in foreshortened images aligns with findings from human echocardiographic studies using GE EchoPac [15], reinforcing the importance of obtaining a true apical view to prevent GLS overestimation. Foreshortening distorts myocardial deformation vectors and alters strain values, underscoring the need for standardized image-acquisition protocols in veterinary echocardiography. This is particularly relevant since foreshortening is a frequent challenge in small animal echocardiography, where optimal transducer positioning is often constrained by patient size and cardiac conformation [17].

The study also found a 15.4% increase in GLS_A4C_ when HR was elevated (*p* = 0.02). This result contrasts with previous assumptions that higher HR may lead to an underestimation of GLS if FR is not adjusted accordingly [16,24]. While prior veterinary and human studies did not observe a significant effect of HR on GLS [8,11], one key difference in the present study is that all dogs were unanesthetized, whereas a previous study used sedated or anesthetized patients, potentially mitigating HR variability [11]. This discrepancy suggests that HR-related changes in GLS may be more pronounced in awake animals, raising important questions about how the physiological state influences myocardial-strain assessments. Further research should explore whether the effect observed here is consistent across different breeds, heart sizes, and clinical conditions.

Adjustments in FR, image zoom, and transducer frequency did not significantly impact GLS_A4C_ values. The slight but non-significant decrease in GLS_A4C_ with higher FR suggests that tracking robustness was preserved above 100 FPS, consistent with prior findings indicating that excessively high FR does not necessarily enhance tracking precision but helps maintain speckle continuity [25]. The interaction between FR and HR is important, especially in small-breed dogs, cats, or dogs with tachycardia, as higher HRs shorten the cardiac cycle and may require higher FRs for accurate speckle tracking. The dogs in our study exhibited a wide range of heart rates, as shown in Table 1. No veterinary studies have evaluated the combined effects of FR and HR on 2D-STE measurements. A pediatric study by Sanchez et al. (2015) indicated that higher FRs reduce observer variability in infants with HRs around 164 bpm, suggesting a need for higher FR at an elevated HR [25]. However, these findings may not directly apply to veterinary medicine due to species differences. Future veterinary studies should investigate the relationship between FR and HR to improve strain accuracy and reproducibility in research and clinical practice. Although zoomed images did not significantly alter GLS_A4C_ measurements, optimizing image width and depth remains important for ensuring high-quality tracking [1,3]. Similarly, transducer frequency adjustments across the tested range had no significant effect on GLS. This aligns with the findings in a human study [26] and is contrary to another [27]. The latter human study did find a significant difference, but the difference was deemed clinically irrelevant by the authors [27]. However, minor variations reported in previous research suggest that the influence of transducer settings may be subtle and vendor-dependent, warranting further investigation in veterinary cardiology [27,28].

In the second cohort, a strong correlation was observed between GLS values derived from the 2D strain and AFI, yet significant differences in 13 of 16 comparisons confirmed that these methods are not interchangeable. To ensure a comprehensive and reproducible assessment of global and regional left ventricular function, the APLAX (three-chamber) view was included alongside the A4C and A2C views, as it visualizes the anterior septal and posterior walls (Figure 1) and completes the standard three-plane acquisition recommended for Bull’s Eye strain mapping in humans [3].

The Bland–Altman analysis demonstrated a systematic bias, with AFI consistently underestimating GLS relative to 2D strain. This is in contrast to findings in human studies, where a similar analysis reported systematic bias in the opposite direction [29]. A potential explanation for this discrepancy is that strain algorithms may interpret myocardial deformation differently in small animal patients compared to humans. Additionally, software updates and algorithm refinements over the past decade could have altered the way these methodologies process strain data, leading to different bias patterns.

A proportional bias was detected in the cardiologist’s AFI analyses, where larger GLS values were associated with greater discrepancies. No bias was seen in the human study [29]. A possibly influencing factor was that the cardiologist, as a routine user, may have employed the two methods with subtle variations.

Intra- and inter-observer variability analysis confirmed high reliability for both methods, with CV values below 9%. This variability is similar to other vendors and STE methods [7,8,22]. However, due to the systematic bias observed between AFI and the 2D strain, a method’s consistency should be prioritized over attempts to interchange results between platforms.

This study examined the impact of technical parameters on GLS measurements using 2D-STE in healthy dogs and examined the interchangeability of GLS values derived from the 2D strain and AFI. The findings provide new insights into how image-acquisition settings influence myocardial deformation assessments and highlight the systemic biases that prevent direct comparability between different strain-analysis methods. While our primary aim was methodological, the GLS values reported here are generally consistent with previous studies in dogs [6,8,11,12], supporting their external validity. These results contribute to the ongoing standardization efforts in veterinary strain imaging and provide important considerations for the clinical interpretation of strain values across various platforms and acquisition conditions.

Despite these findings, several limitations must be acknowledged. The relatively small sample size may have reduced the statistical power of some analyses, and demographic factors such as age, breed, and sex were not specifically examined, although previous studies have investigated these influences [8,12]. The study cohort predominantly consisted of small and miniature breed dogs, as these were the individuals presenting for voluntary screening and allowed for acquiring high-quality apical views without sedation. While this enhances the methodological consistency of the study, it may limit generalizability to larger breeds, which can make apical imaging more challenging. Future studies should aim to include a broader range of body sizes to assess potential breed-related differences in 2D-STE feasibility and measurement variability. All comparisons in the second cohort were performed on the same images, minimizing intra-individual variability. Another limitation is that only high-quality echocardiographic images were analyzed, which may not reflect real-world clinical conditions where image quality varies considerably, particularly in patients with heart disease. This study also relied on a single vendor for both image acquisition and offline analysis, limiting the generalizability of results to other echocardiographic platforms. Future research should assess the robustness of 2D-STE across different ultrasound vendors and under the suboptimal imaging conditions commonly encountered in clinical practice.

In analyzing both cohorts, some challenges were encountered during image analysis. Manual adjustment of the ROI was necessary for all the analyzed images. The algorithm’s width selection was set too wide, resulting in tracking an area larger than the myocardium, including the pericardium. This adjustment process was time-consuming and introduced a risk of obtaining unreliable strain results. It is important to set the width correctly to include the entire myocardium. GLS is higher in the endocardium and lower in the epicardium [14]. Therefore, if a too-narrow ROI is chosen, including only the endocardial layer, it can overestimate strain values. On the other hand, if the ROI is too wide, encompassing the pericardium, the strain values will most likely be underestimated [3,14].

## 5. Conclusions

This study confirms that GLS measurements are significantly influenced by image foreshortening and increased HR, leading to GLS overestimation. In contrast, changes in FR, image zoom, and transducer frequency did not significantly alter GLS values. Moreover, due to systemic bias, the 2D strain and AFI cannot be used interchangeably despite their strong correlation for GLS measurements. A proportional bias was detected in the cardiologist’s AFI analyses, with greater discrepancies at higher strain values. The low intra- and inter-observer variabilities observed for both methods reinforce their reliability. However, their systematic differences highlight the importance of method-specific standardization in clinical and research settings. These findings emphasize the need for optimized image-acquisition protocols, careful methodological consistency, and standardized reporting of strain-analysis techniques in veterinary cardiology. Future studies should further investigate the impact of different vendor platforms, clinical disease states, and patient-specific factors on 2D-STE measurements to refine its clinical application in veterinary medicine.

## Figures and Tables

**Table 1:** Demographic data from the healthy dogs included in the first and second cohorts.

	Cohort 1	Cohort 2
Patients included	16	10
Age (years)	2.9 (1.3–4.8)	2.0 (1–4)
Breed		
CKCS	11 (68.75%)	5 (50%)
Miniature bull terrier	1 (6.25%)	0 (0%)
Broholmer	1 (6.25%)	0 (0%)
Bull terrier	1 (6.25%)	0 (0%)
Shih Tzu	0 (0%)	1 (10%)
Chihuahua	0 (0%)	1 (10%)
Danish–Swedish farm dog	0 (0%)	1 (10%)
Jack Russell	0 (0%)	1 (10%)
Small mix	2 (12.5%)	0 (0%)
Medium mix	0 (0%)	1 (10%)
Heart rate (bmp)	128 (85–159)	112 (91–146)
Sex		
Female	12 (75%)	7 (70%)
Male	4 (25%)	3 (30%)
Weight (kg)	11.7 (4.6–45.0)	8.6 (3.7–21.9)
Body Condition Score (Grade 1-9)	4.8 (4–7)	4.6 (4–5)

Data presented is n (%) or mean (range). Abbreviations: BPM, Beats per minute, CKCS, Cavalier King Charles Spaniel.

**Table 2:** Statistics of the 2D strain measurements in the first cohort containing 16 healthy dogs. n = number of dogs analyzed. *p*-value of *t*-test shown compared to baseline (A4C).

Parameter	n	Mean ± SD	Min.	Max.	*p*-Value
GLS_A4C_	16	−19.19% ± 4.11%	−12.90%	−25.70%	-
GLS_A4C-Zoom_	16	−19.67% ± 4.35%	−12.90%	−30.30%	0.57
GLS_A4C↑Frame rate_	16	−18.07% ± 2.61%	−14.10%	−22.30%	0.15
GLS_A4C-Foreshortening_	13	−21.32% ± 3.51%	−16.50%	−30.20%	<0.01
GLS_A4C↑HR_	11	−22.15% ± 4.08%	−17.80%	−30.30%	0.02
GLS_A4C-Frequency_	15	−19.35% ± 3.46%	−12.80%	−26.70%	0.47

Abbreviations: GLS, global longitudinal strain; A4C: apical four-chamber; Zoom: zoomed; HR, heart rate.

**Table 3:** 2D strain and AFI analyses in the second cohort included 10 healthy dogs. Results shown for GLS obtained from the three left apical views, together with the individual GLS results for the apical long axis, four-chamber, and two-chamber views, respectively. *p*-value for the *t*-test and the r-value for the Pearson correlation test, comparing the strain measurements obtained by 2D strain and AFI, were presented.

	2D Strain	AFI	*p*-Value	r-Value
GLS	−20.04% ± 2.88%	−17.67% ± 1.61%	<0.01	0.87
GLS_APLEX_	−20.17% ± 4.40%	−17.49% ± 2.72%	0.03	0.61
GLS_A4C_	−20.27% ± 3.08%	−18.26% ± 1.66%	<0.01	0.83
GLS_A2C_	−19.70% ± 3.18%	−17.24% ± 2.38%	<0.01	0.87

Data presented are mean ± SD, a *p*-value, and an r-value. Abbreviations: 2D strain, quantitative analysis for 2D strain; AFI, automated function imaging; GLS, global longitudinal strain. APLAX: apical long axis; A4C: apical four-chamber; A2C: apical two-chamber.

**Table 4:** Intra- and inter-observer variability between quantitative analysis for 2D strain and automated function imaging for the GLS for all three views.

	2D Strain	AFI
Intra-observer repeatability CV		
GLS	3.86%	2.61%
GLS_APLAX_	7.37%	3.91%
GLS_A4C_	8.17%	4.06%
GLS_A2C_	7.33%	3.46%
Inter-observer repeatability CV		
GLS	4.24%	5.66%
GLS_APLAX_	8.71%	8.29%
GLS_A4C_	8.08%	5.26%
GLS_A2C_	6.45%	6.77%

Abbreviations: 2D strain, quantitative analysis for 2D strain; AFI, automated function imaging; GLS, global longitudinal strain; CV, coefficient of variance; APLAX: apical long axis; A4C: apical four-chamber; A2C: apical two-chamber.

## Data Availability

Data were not disclosed due to ongoing research.

## Abbreviations

The following abbreviations are used in this manuscript:

2D-STE	Two-dimensional speckle tracking echocardiography
2D Strain	Quantitative analysis for 2D strain
A2C	Automated function imaging
A4C	Apical two-chamber
AFI	Apical four-chamber
APLAX	Apical long axis
CV	Coefficient of variance
ECG	Electrocardiography
FR	Frame rate
FPS	Frames per second
GLS	Global longitudinal strain
HR	Heart rate
MHz	Megahertz
ROI	Region of interest
